# Heart failure hospitalization risk associated with use of two classes of oral antidiabetic medications: an observational, real-world analysis

**DOI:** 10.1186/s12933-017-0575-x

**Published:** 2017-07-31

**Authors:** Santosh Gautam, Abiy Agiro, John Barron, Thomas Power, Harry Weisman, Jeff White

**Affiliations:** 1 HealthCore, Inc., 123 Justison St, Suite 200, Wilmington, DE 19801 USA; 2 AIM Specialty Health, Chicago, IL USA; 3 Anthem, Inc., Indianapolis, IN USA

**Keywords:** SGLT2, DPP4, Type 2 diabetes, OAD, Heart failure, Claims analysis

## Abstract

**Background:**

Newer oral antidiabetic drug classes are expanding treatment options for type 2 diabetes mellitus (T2DM); however, concerns remain. The objective was to assess relative risk of heart failure hospitalization of sodium–glucose co-transporter-2 (SGLT2) and dipeptidyl peptidase-4 (DPP4) inhibitors in T2DM patients.

**Methods:**

This retrospective observational study used a national commercially insured claims database. Adults (>18 years) with T2DM newly starting SGLT2 or DPP4 medication between April 2013 and December 2014 were included. Depending on their index fill, patients were grouped into either SGLT2 or DPP4 medication class cohorts. The primary outcome was hospitalization for heart failure and the risk was assessed using Cox regression models. Propensity score matching (1:2 ratio) was used to adjust for potential confounders. Analyses were also stratified by the presence of baseline diabetes complication and age (<65 vs 65+).

**Results:**

The matched cohort included 4899 SGLT2 and 9798 DPP4 users. The risk of heart failure hospitalization was lower among SGLT2 users in comparison with matched DPP4 users (2.0% SGLT2 vs 3.1% DPP4; adjusted hazard ratio [aHR] 0.68; 95% confidence interval [CI] 0.54–0.86; p = .001). However, the stratified analyses revealed no risk difference among the majority of the analyzed patients, i.e., those aged <65, which comprised 85% of the matched cohort (aHR = 0.78; 95% CI 0.57–1.05; p = .09), and those without prior complication, which comprised 69% of matched cohort (aHR = 0.83; 95% CI 0.54–1.27; p = 0.40).

**Conclusions:**

In this real-life analysis, the rate of hospitalizations for heart failure was significantly lower for patients initiating an SGLT2 compared with a DPP4 medication, specifically among older patients and those with diabetes complication.

**Electronic supplementary material:**

The online version of this article (doi:10.1186/s12933-017-0575-x) contains supplementary material, which is available to authorized users.

## Introduction

The rising prevalence of type 2 diabetes mellitus (T2DM) is concerning because of significant morbidity and mortality associated with the condition [1, 2]. The risk of heart failure alone is four- to fivefold higher among patients with diabetes compared with those without the condition [3], and even more so among men and patients older than 65 years [4]. Additionally, patients with diabetes face a higher risk of a recurrent heart failure event and worse outcomes, compared with people without diabetes [5]. Current Food and Drug Administration guidelines recommend cardiovascular risk evaluation for drugs intended to treat T2DM [6].

Recently, two relatively new classes of oral antidiabetic drugs (OADs)—sodium–glucose co-transporter 2 (SGLT2) inhibitors and dipeptidyl peptidase-4 (DPP4) inhibitors—have received attention regarding their effect on cardiovascular outcomes [7–13]. SGLT2 inhibitors are the latest addition to the T2DM treatment armamentarium and are recommended as second-line treatment after metformin, as are the DPP4 inhibitors [14]. The EMPA-REG OUTCOME clinical trial found no cardiovascular risk concerns with empagliflozin, an SGLT2 inhibitor [13]. In fact, the study showed an improvement in cardiovascular prognosis, as evidenced by a lower rate of composite cardiovascular events compared with placebo, and a lower rate of heart failure-associated hospital admissions [13]. Recently the Food and Drug Administration approved empagliflozin to reduce cardiovascular death among patients with T2DM and cardiovascular diseases [15]. Since the study was published, there has been growing interest to establish whether the beneficial impact is a class-level effect, but limited information is available.

Conversely, cardiovascular outcome studies with DPP4 inhibitors have provided conflicting results. The SAVOR-TIMI 53 trial demonstrated the DPP4 inhibitor saxagliptin was associated with a higher rate of heart failure hospitalization than placebo [10]. However, heart failure hospitalization rates for sitagliptin and alogliptin, also DPP4 inhibitors, were comparable to placebo in the TECOS [9] and EXAMINE trials [12], respectively. Two recently published observational studies concluded DPP4 inhibitors had similar risks of heart failure as other antidiabetic medications [7, 11]. Some studies even have suggested that DPP4 inhibitors may reduce the risk of heart failure [16–18]. Amid those uncertainties, the Food and Drug Administration has warned that two DPP4 inhibitors, saxagliptin and alogliptin, may increase the risk of heart failure, especially in patients who already have cardiovascular or kidney disease [19].

In light of the limited beneficial evidence on SGLT2 inhibitors and potential risk concerns with some of the DPP4 inhibitors, we undertook this study to examine how these two newer drug classes compare with each other in terms of associated risk of hospitalization for heart failure. This is the first study reporting a direct comparison of SGLT2 and DPP4 medication classes on the risk of heart failure hospitalization.

## Methods

### Data source

A retrospective observational study was conducted using medical and pharmacy claims data from October 2012 to October 2016. The data were obtained from the HealthCore Integrated Research Environment^SM^, which consists of claims and eligibility files from 14 commercial health plans with members geographically dispersed across the United States. This study was exempt from Institutional Review Board review as the researchers only accessed a non-identifiable dataset in full compliance with relevant provisions of the Health Insurance Portability and Accountability Act of 1996.

### Patient cohort identification

The study included adults (aged 18 years or older) who filled a new prescription for SGLT2 or DPP4 medication classes between April 2013 and December 2014. The earliest prescription fill date was defined as the index date. Depending on their index prescription fill, patients were assigned into either SGLT2 or DPP4 medication class cohorts. As our study aim was to conduct a class-level analysis, we did not analyze individual agents. In addition, sample sizes were too small for some agents to conduct a meaningful analysis. The distribution of agents on index date for each medication class is provided in a supplemental table (Additional file 1: Table S1).

We restricted the cohorts to new users only by excluding those who filled a prescription for SGLT2, DPP4, or glucagon-like peptide-1 (GLP1) inhibitors (to avoid confounding due to GLP1 inhibitors, which, like DPP4 inhibitors, are also incretin-based therapy) during the 6 months prior to index date. Undertaking a new user study design approach in an observational study such as ours is important as prevalent users could introduce substantial bias [20]. All patients had at least 6 months of continuous medical and pharmacy insurance coverage prior to the index date for identification of baseline characteristics. Patients were excluded if they had a diagnosis of type 1 diabetes as neither medication class is indicated for type 1 patients.

### Primary outcome and follow up

The primary outcome was hospitalization for heart failure. Inpatient claims were searched for *International Classification of Diseases, Ninth* or *Tenth Revision*, diagnosis codes (ICD-9: 428, ICD-10: I50) to identify patients who encountered a heart failure hospitalization. Patients were followed from index date through October 31, 2016 looking for the outcome occurrence and were censored at the end of the study period or the end of health plan enrollment, whichever came first.

### Covariates

We obtained the following baseline characteristics for each patient based on claims information during the 6 months prior to index date: age, gender, comorbidity (defined by Deyo–Charlson comorbidity index score [21]), provider specialty on index fill, diabetes complications [22] (cardiovascular, neuropathy, nephropathy, retinopathy, peripheral vascular, cerebrovascular, and metabolic complications), history of adverse events (heart failure, renal diseases, urinary tract infection, hypoglycemia, ketoacidosis, acute pancreatitis), prior OAD exposure (metformin, sulfonylureas, thiazolidinediones, OAD combinations, meglitinide, alpha-glucosidase inhibitors), medication possession ratio [23] of any OAD (as a proxy for persistent exposure to antidiabetic therapy), insulin use, and other concomitant medications (angiotensin converting enzyme inhibitors/angiotensin receptor blockers, diuretics, mineralocorticoids, beta blockers, statins, and other cardiovascular medications). Clinical conditions were identified using ICD-9 or ICD-10 codes and medications were identified using General Product Identifier codes.

### Statistical analysis

Differences in baseline characteristics between the cohorts were compared using *t* test for continuous and χ^2^ test for categorical variables. Additionally, we used standardized differences to compare those characteristics. Standardized differences were defined as differences between cohort means or proportion relative to the pooled standard deviation. Standardized differences are a useful measure as they are not sensitive to sample size, unlike traditional tests of statistical significance, and a difference of 10% or more is generally considered meaningful [24, 25]. Unadjusted difference in the outcome was estimated using the Kaplan–Meier analysis and Cox regression.

To account for the baseline differences between the cohorts, we considered the propensity score matching technique. A propensity score for SGLT2 (or DPP4) treatment was calculated using a logistic regression model using all the covariates described above. A matched cohort was created by matching a patient in SGLT2 cohort with two patients in DPP4 cohort based on their propensity scores using a greedy matching algorithm [26]. The balance in baseline characteristics between the two matched cohorts was assessed using standardized differences. The balance was also visually assessed using boxplots of propensity scores. Finally, we ran a Cox regression model within the matched cohorts to calculate the adjusted risks of heart failure hospitalization.

### Consistency analysis

Propensity score matching is a widely used technique known for reducing bias; however, it may impair generalizability by leaving out a substantial number of unmatched patients from the analysis. To overcome this issue, we performed consistency analyses using two different analytical approaches—inverse probability of treatment weighting (IPTW) and marginal structural modeling (MSM). For IPTW analysis, we use the method outlined by Austin [27]. First, we calculated inverse probability of treatment weights using a propensity model that included all of the covariates listed above. The balance in baseline characteristics between two cohorts after applying IPTWs was verified. Then, the IPTWs were used in a weighted Cox proportional hazard model to estimate the adjusted hazard ratio (HR) for heart failure hospitalization.

For the MSM analysis, we followed the method outlined by Fewel et al. [28]. MSMs use inverse probability of treatment weights that take into account the probability of being on treatment as well as the probability of being uncensored once the follow-up begins. MSMs are known for producing unbiased estimates by accounting for time varying confounding or informative censoring in addition to addressing baseline differences between study cohorts [29]. The weights were calculated using baseline covariates along with time-varying GLP1 and insulin use. The calculated weights were used in the Cox regression model to estimate the adjusted hazard ratios.

### Sensitivity and subgroup analyses

To reduce potential biases due to differential exposure and loss to follow-up, we performed two separate sensitivity analyses: (a) by restricting the cohort to those who had at least 90 days of exposure to index medication class, and (b) by restricting the cohort to those who had at least 24 months of continuous health plan enrollment after the index date. Additionally, we carried out subgroup analyses by age (<65 vs ≥65) and history of any diabetes complication (yes vs no).

All treatment effects estimates were based on an intent-to-treat principle and a p value <.05 was deemed statistically significant. Statistical analyses were conducted using Stata 13.0 (StataCorp) and SAS Enterprise Guide 7.1 (SAS, Inc).

## Results

### Patient characteristics

We identified 5467 patients in the SGLT2 and 32,060 in the DPP4 cohorts who were eligible for the study. After matching, there were 4899 members in the SGLT2 cohort and 9798 in the DPP4 cohort.

Before matching, there were considerable differences between the study cohorts. Compared with patients in the DPP4 cohort, those in the SGLT2 cohort were younger (mean age 55 years SGLT2 vs 59 years DPP4, p < .001; Table 1). A higher proportion of patients in the SGLT2 cohort were women (46.0% SGLT2 vs 42.9% DPP4, p < .001) and had fewer comorbidities (mean baseline comorbidity score 1.43 SGLT2 vs 1.69 DPP4, p < .001). A lower proportion of patients in the SGLT2 cohort had complications of diabetes at baseline, compared with the DPP4 cohort (32.6% SGLT2 vs 40.0% DPP4, p < .001). Additionally, a lower proportion of patients in the SGLT2 cohort had a history of heart failure (2.1% SGLT2 vs 5.1% DPP4, p < .001).

**Table 1:** Comparison of baseline demographic and clinical characteristics in two study cohorts before and after matching

	Overall unmatched cohorts			Matched cohorts		
	DPP4 n = 32,060	SGLT2 n = 5467	p value* (standardized difference, %)	DPP4 n = 9798	SGLT2 n = 4899	p value* (standardized difference, %)
Age at index date (mean, SD)	59.0 (12.5)	54.6 (10.0)	<.001 (39%)	55.1 (0.1)	54.9 (0.1)	.19 (2%)
Age categories, n (%)			<.001			.68
18–44	3911 (12.2)	871 (15.9)	(11%)	1591 (16.2)	757 (15.5)	(2%)
45–54	7713 (24.1)	1689 (30.9)	(15%)	2942 (30.0)	1484 (30.3)	(1%)
55–64	10,386 (32.4)	2185 (40.0)	(16%)	3832 (39.1)	1937 (39.5)	(1%)
≥65	10,050 (31.4)	722 (13.2)	(45%)	1433 (14.6)	721 (14.7)	(0%)
Gender, n (%)			<.001			.73
Female	13,744 (42.9)	2513 (46.0)	(6%)	4450 (45.4)	2210 (45.1)	(1%)
Comorbidity						
DCI, mean (SD)	1.69 (1.5)	1.43 (1.4)	<.001 (20%)	1.4 (0.01)	1.4 (0.02)	.54 (1%)
DCI categories, n (%)			<.001			.24
0	2483 (7.7)	330 (6.0)	(7%)	721 (7.4)	322 (6.6)	(3%)
1	18,075 (56.4)	3509 (64.2)	(16%)	6276 (64.1)	3162 (64.5)	(1%)
2	5613 (17.5)	1014 (18.6)	(3%)	1729 (17.7)	849 (17.3)	(1%)
3	5889 (18.4)	614 (11.2)	(20%)	1072 (10.9)	566 (1.6)	(2%)
Provider specialty on index fill, n (%)			<.001			.96
Endocrinologist	3327 (10.4)	1114 (20.4)	(28%)	1425 (14.5)	710 (14.5)	(0%)
PCP	23,215 (72.4)	3382 (61.9)	(23%)	6581 (67.2)	3283 (67.0)	(0%)
Other	5518 (17.2)	971 (17.8)	(1%)	1792 (18.3)	906 (18.5)	(1%)
Diabetes complications, n (%)						
Any	12,816 (40.0)	1784 (32.6)	<.001 (15%)	3032 (31.0)	1561 (31.9)	.26 (2%)
Cardiovascular	6635 (20.7)	726 (13.3)	<.001 (20%)	1318 (13.5)	671 (13.7)	.68 (1%)
Neuropathy	4170 (13.0)	751 (13.7)	.14 (2%)	1148 (11.7)	616 (12.6)	.13 (3%)
Nephropathy	3663 (11.4)	312 (5.7)	<.001 (21%)	544 (5.6)	290 (5.9)	.36 (2%)
Retinopathy	2165 (6.8)	336 (6.2)	.10 (3%)	548 (5.6)	274 (5.6)	1.00 (0%)
Peripheral vascular	1886 (5.9)	191 (3.5)	<.001 (11%)	345 (3.5)	180 (3.7)	.64 (1%)
Cerebrovascular	1580 (4.9)	129 (2.4)	<.001 (14%)	273 (2.8)	125 (2.6)	.41 (2%)
Metabolic	204 (0.6)	35 (0.6)	0.97 (0%)	49 (0.5)	28 (0.6)	.57 (1%)
History of adverse events, n (%)						
Renal	2618 (8.2)	155 (2.8)	<.001 (24%)	297 (3.0)	155 (3.2)	.66 (1%)
UTI	2106 (6.6)	279 (5.1)	<.001 (6%)	509 (5.2)	258 (5.3)	.85 (0%)
CHF	1641 (5.1)	115 (2.1)	<.001 (16%)	225 (2.3)	112 (2.3)	.97 (0%)
Hypoglycemia	886 (2.8)	157 (2.9)	0.65 (1%)	248 (2.5)	130 (2.7)	.66 (1%)
Ketoacidosis	101 (0.3)	13 (0.2)	0.34 (2%)	22 (0.2)	12 (0.2)	.81 (0%)
Pancreatitis	98 (0.3)	30 (0.6)	.004 (4%)	36 (0.4)	14 (0.3)	.42 (1%)
Prior OAD use, n (%)						
Any	24,394 (76.1)	4183 (76.5)	.50 (1%)	7496 (76.5)	3721 (76.0)	.46 (1%)
Metformin	19,700 (61.5)	3431 (62.8)	.07 (3%)	6105 (62.3)	3084 (63.0)	.45 (1%)
Sulfonylurea	10,576 (33.0)	1720 (31.5)	.03 (3%)	3056 (31.2)	1575 (32.2)	.24 (2%)
TZD	1880 (5.9)	404 (7.4)	<.001 (6%)	649 (6.6)	338 (6.9)	.53 (1%)
Combination OAD	1001 (3.1)	272 (5.0)	<.001 (9%)	421 (4.3)	213 (4.4)	.89 (0%)
Meglitinide	370 (1.2)	58 (1.1)	.55 (1%)	91 (0.9)	52 (1.1)	.44 (1%)
Alpha-glucosidase inhibitor	109 (0.3)	19 (0.4)	.93 (0%)	25 (0.3)	16 (0.3)	.44 (1%)
MPR of any OAD, mean (SD)	0.44 (0.4)	0.47 (0.5)	<.001 (10%)	0.45 (0.004)	0.46 (0.005)	.14 (3%)
Insulin, n (%)	3237 (10.1)	1481 (27.1)	<.001 (45%)	1848 (18.9)	920 (18.8)	.91 (0%)
Other medications, n (%)						
Cardiovascular drugs	4096 (12.8)	445 (8.1)	<.001 (17%)	869 (8.9)	403 (8.2)	.19 (2%)
Statin	18,578 (58.0)	3017 (55.2)	<.001 (6%)	5307 (54.2)	2696 (55.0)	.32 (2%)
ACE/ARB	14,856 (46.3)	2418 (44.2)	.004 (4%)	4211 (43.0)	2139 (43.7)	.43 (1%)
Diuretic	10,991 (34.3)	1774 (32.5)	.008 (4%)	3063 (31.3)	1585 (32.4)	.18 (2%)
Spironolactone	709 (2.2)	114 (2.1)	0.56 (1%)	172 (1.8)	104 (2.1)	.12 (2%)
Beta blocker	9171 (28.6)	1292 (23.6)	<.001 (11%)	2250 (23.0)	1159 (23.7)	.35 (2%)

*ACE/ARB* angiotensin converting enzyme inhibitor/angiotensin receptor blocker, *CHF* coronary heart failure, *DCI* Deyo–Charlson comorbidity index, *DPP4* dipeptidyl peptidase-4, *MPR* medication possession ratio, *OAD* oral antidiabetic drug, *PCP* primary care provider, *SD* standard deviation, *SGLT2* sodium–glucose co-transporter 2, *TZD* thiazolidinediones, *UTI* urinary tract infection

* p values were derived from χ^2^ tests for categorical variables and t tests for continuous variables. Comparison group was DPP4

However, after matching, the characteristics were well balanced as none of them remained significantly different between the two matched cohorts (Table 1). The balance was also confirmed through visual assessment (Additional file 2: Figure S1).

### Follow up and outcome

In the overall unmatched cohort, the median length of follow up for the SGLT2 cohort was 23.0 months (interquartile range 10.6–28.3 months, maximum 42.7 months; Table 2), whereas it was 23.8 months for the DPP4 cohort (interquartile range 9.7–32.0 months, maximum 42.9 months). The follow-up period in the matched-cohorts was comparable to that of overall unmatched cohorts. In terms of exposure, the average number of fills and average days covered were comparable in both the unmatched and matched cohorts.

**Table 2:** Comparison of unadjusted risk of heart failure hospitalization in two study cohorts before and after matching

	Overall unmatched cohorts		Matched cohorts	
	DPP4 n = 32,060	SGLT2 n = 5467	DPP4 n = 9798	SGLT2 n = 4899
Follow up length (months)				
Median	23.8	23.0	24.0	22.9
25th, 75th percentiles	9.7, 32.0	10.6, 28.3	10.5, 32.1	10.5, 30.8
Maximum	42.9	42.7	42.9	42.7
Exposure to index medication class				
Average number of fills	10.2	11.3	10.4	11.2
Average number of days covered	359	357	365	354
Patients w/heart failure hospitalization	1734	109	307	96
Proportion of patients w/heart failure hospitalization (%)	5.4	2.0	3.1	2.0
Person-years of follow up, n	58,018	9192	17,981	8228
Event rate (95% CI) per 100 person years	2.99 (2.85–3.13)	1.19 (0.98–1.43)	1.71 (1.53–1.91)	1.17 (0.96–1.43)
HR (95% CI), unadjusted	0.39 (0.32–0.48); p < .001		0.68 (0.54–0.86); p = .001	

*CI* confidence interval, *DPP4* dipeptidyl peptidase-4, *HR* hazard ratio, *SGLT2* sodium–glucose co-transporter 2

**Fig. 1:**
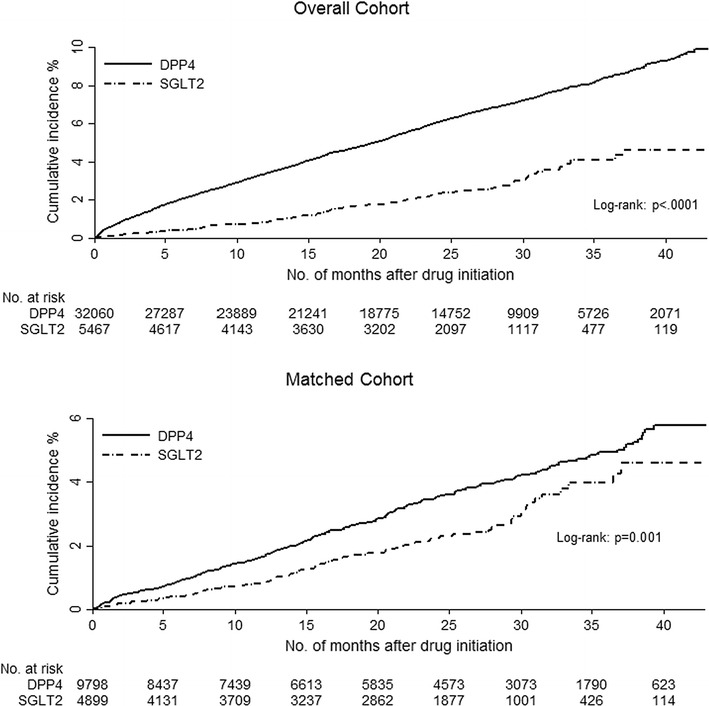
Cumulative incidence of heart failure hospitalization in two study cohorts before and after matching

Table 2 lists and Fig. 1 shows the difference in outcomes between the unmatched and matched cohorts. In the overall unmatched cohorts, patients in the SGLT2 cohort had a lower rate of heart failure hospitalization than patients in the DPP4 cohort (109 patients, 2.0% SGLT2 vs 1734 patients, 5.4% DPP4) and the unadjusted HR for the SGLT2 cohort was 0.39 (95% CI 0.32–0.48; p < .001) compared with the DPP4 cohort (Table 2).

**Fig. 2:**
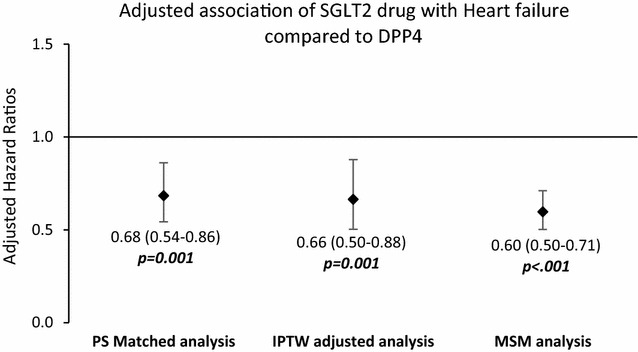
Adjusted hazard ratios for heart failure hospitalization using different analytical methods in two study cohorts

In the matched cohort, the difference in the rate of heart failure hospitalization decreased slightly (96 patients, 2.0% SGLT2 vs 307 patients, 3.1% DPP4). The associated adjusted risk indicated that patients in the SGLT2 cohort were 32% less likely to have an admission for heart failure than those in the DPP4 cohort (adjusted HR [aHR] 0.68; 95% CI 0.54–0.86; p = .001). Consistency analyses on all patients using IPTW adjusted method yielded an aHR of 0.66 (95% CI 0.50–0.88; p = .001; Fig. 2) whereas the MSM model yielded an aHR of 0.60 (95% CI 0.50–0.71; p < .001) for the SGLT2 cohort compared with the DPP4 cohort.

### Subgroup analyses

In the matched cohorts of patients aged 65 years or older, the proportion of patients with heart failure hospitalization was lower in the SGLT2 cohort compared with the matched patients in DPP4 cohort (4.7% SGLT2 vs 9.1% DPP4; aHR 0.60; 95% CI 0.41–0.87; p = .008; Additional file 3: Table S2). Among those younger than 65 years, the risk of heart failure was slightly lower in the SGLT2 cohort compared with the DPP4 cohort; however, the association was not significant (aHR 0.77; 95% CI 0.57–1.05; p < .09; Additional file 3: Table S2, Additional file 4: Figure S2).

Similarly, the proportion of admissions for heart failure was lower in the SGLT2 cohort compared with the DPP4 cohort among matched cohorts of patients with diabetes complication (4.5% SGLT2 vs 6.9% DPP4; aHR 0.68; 95% CI 0.52–0.90; p = .006; Additional file 3: Table S2). Among the matched cohorts without diabetes complication, a lower proportion of members in the SGLT2 cohort experienced the event; however, the risk was not statistically significant (aHR 0.83; 95% CI 0.54–1.27; p = 0.40; Additional file 3: Table S2, Additional file 5: Figure S3). We separately assessed and confirmed that age 65 years or older and established diabetes complication are independently associated with heart failure hospitalization.

### Sensitivity analyses

After restricting the matched cohorts to those who had at least 90 days of exposure to the index class drug, the heart failure hospitalization rate still remained lower for the SGLT2 cohort compared with the DPP4 cohort (61 patients, 1.7% in SGLT2 vs. 182 patients, 2.5% in DPP4) with aHR 0.74 (95% CI 0.55–0.98, p = 0.038; Additional file 6: Table S3). We observed a similar trend in the matched cohorts with at least 24 months of post-index enrollment in the health plan. There were 60 patients (2.7%) in the SGLT2 cohort and 180 patients (4.1%) in the DPP4 cohort who experienced a heart failure hospitalization event (aHR = 0.72, 95% CI 0.54–0.97; p = 0.030).

## Discussion

The goal of this study was to compare the risk of heart failure hospitalization between two relatively new classes of oral antidiabetic agents, DPP4 and SGLT2 inhibitors, among patients with T2DM in a real-world setting. In the propensity score-matched cohorts, we observed a lower adjusted risk of heart failure hospitalization among patients taking SGLT2 inhibitors than those taking DPP4 inhibitors. This result changed slightly and remained significant in our consistency analyses in all patients (including unmatched patients) using different statistical approaches as well as in the sensitivity analyses around medication exposure and follow-up durations. However, our subgroup analyses revealed that there was no difference in heart failure hospitalization risks between the two medication classes among the majority of the analyzed patients, i.e., those younger than 65 years (85.3% of matched cohort) and also in patients without a prior history of diabetes complications (68.7% of matched cohort).

### DPP4 inhibitors and heart failure

Heart failure risks associated with several DPP4 inhibitors have been assessed in randomized clinical trials, meta-analyses, and observational studies, producing conflicting results ranging from reduced risk [3, 17, 18], to no risk [7, 9, 11, 12, 30–32], to high risk [8, 16, 30, 33–35] of heart failure. Such discrepant findings may have stemmed primarily from different comparator groups across these studies as well as differences in study design and population selection. For this very reason, it was difficult to make a head-to-head comparison of our results with those previous studies. However, it is noteworthy that no prior studies directly compared DPP4 inhibitors with SGLT2 inhibitors, and our finding that at a class level DPP4 inhibitors are associated with higher risk of heart failure hospitalization than SGLT2 inhibitors will be an important addition to the literature.

### SGLT2 inhibitors and heart failure

On the other hand, there are few published studies examining the heart failure risk associated with SGLT2 medications, and they have reported more consistent results. For example, the EMPA-REG study, the first randomized clinical trial to examine an SGLT2 inhibitor, found that empagliflozin was associated with lower risks of heart failure hospitalization compared with placebo (HR 0.65; 95% CI 0.50–0.85) [13]. Another recently published clinical trial reported a lower risk of heart failure hospitalization associated with canagliflozin, another SGLT2 inhibitor, compared with placebo (HR 0.67; 95% CI 0.52, 0.87) [36]. Similar findings were reported in a multinational observational study comparing class level effect of SGLT2 inhibitors to other glucose-lowering drugs with a reported HR of 0.61 (95% CI 0.51–0.73) [37]. Those findings are consistent with our results; however, such comparisons warrant some caution as our comparator group was DPP4 rather than placebo or any glucose-lowering drugs. Additionally, there were differences in patient risk profile and follow-up time. Nevertheless, our finding is intriguing in light of the overwhelming interest around the reduction of heart failure risks associated with SGLT2 inhibitors.

### Potential class level effect and underlying mechanism

Since publication of the EMPA-REG trial, two important questions have been gaining ground: first, whether the protective effect of SGLT2 on heart failure is a class-level effect [38, 39]; and second, speculation as to the possible underlying mechanism. Recently published studies by Neal et al. and Kosiborod et al. point towards a potential class-level effect of SGLT2 inhibitors [36, 37]. Our study also shows lower rate of heart failure hospitalization associated with each drugs in the SGLT2 class and hence serves as an additional piece of evidence to support the earlier findings (see Additional file 1: Table S1). The results from ongoing trial examining dapagliflozin (Dapagliflozin Effect on CardiovascuLAR Events [40]) remain crucial for establishing the class-level effect of SGLT2 inhibitors on risk of heart failure hospitalization, although a recent meta-analysis suggests that the effect of dapagliflozin on heart failure hospitalization is comparable to empagliflozin [41].

Regarding the question of underlying mechanism, studies that have examined the cardiovascular effect of DPP4 inhibitors are not able to establish a direct link with cardiac or endothelial effect [16, 42]. On the other hand, studies examining beneficial cardiovascular effect of SGLT2 inhibitors have pointed out several plausible pathways such as glycemic control, body weight reduction, lowering in blood pressure, and reduction in albuminuria, but it is still not clearly understood [36, 39, 43, 44]. Studies mostly agree that the observed cardiovascular effect is unlikely to be coming from a direct effect of SGLT2 on cardiac function [43–46]. Exploring an in-depth understanding of the underlying mechanism is necessary to establish a more robust evidence base. Ongoing clinical trials evaluating cardiovascular effects of SGLT2 inhibitors may shed additional light on it in the future [47].

To the best of our knowledge, this is the first observational study comparing heart failure risks associated with the SGLT2 and DPP4 medication classes. The findings of this study have important implications. These results could prove to be useful to clinicians providing care to patients with T2DM at risk for heart failure and to the patients themselves, as well as to regulatory agencies and professional societies [48, 49]. The strengths of our study include a large sample size, approximately 2 years of average follow up, findings based on equally balanced treatment cohorts, and assessment of the robustness of the results using multiple statistical methods followed by several subgroup and sensitivity analyses.

### Limitations

Several limitations should be considered when interpreting the results of this study. First, this is a class-level analysis of risk differences and should not be extrapolated to agent-level differences. Selection bias may have been present as this study was a comparison of non-randomized treatment groups. We attempted to minimize such biases by balancing our cohorts through propensity score matching using an array of potential confounders. Results were also further verified through consistency analyses that included all patients using IPTW and MSM methods. Yet, residual bias may have remained due to unmeasured confounding from factors such as body mass index, smoking status, and other behavioral health aspects. This information is important in assessing diabetes outcomes, but is rarely available in claims data. Claims data also lack information on diabetes onset or duration, which could potentially affect the outcomes discussed. Additionally, while it is possible to determine that prescriptions for OADs were filled, it is uncertain whether patients took the medication as dispensed. Another potential limitation could be the differential follow-up time in the two cohorts, which could have affected the exposure time and outcomes. To address this limitation, we conducted a sensitivity analysis using a marginal structural model that allowed accounting for censoring and adjusting for time varying confounding [50] and found the results to be similar in terms of trend directionality and statistical significance.

## Conclusions

In this real-world analysis of T2DM patients, our findings suggest that SGLT2 inhibitors are associated with lower risk of heart failure hospitalization than DPP4 inhibitors, specifically among patients older than 65 years of age or those with a prior history of diabetic complications. Future studies could focus on comparisons of the SGLT2 class with other antidiabetic drug classes to further confirm the potential class level effect of SGLT2 drugs. Additionally, future studies could also compare individual agents and examine other cardiovascular outcomes, such as myocardial infarction and stroke, to establish more robust evidence around those therapies.

## Additional files

**Additional file 1: Table S1.** Breakdown of individual agents in each drug class.

**Additional file 2: Figure S1.** Visual assessment of propensity scores—before and after matching.

**Additional file 3: Table S2.** Heart failure hospitalization—subgroup analysis by age and history of cardiovascular disease.

**Additional file 4: Figure S2.** Cumulative incidence of heart failure hospitalization among patients younger than 65 years (before and after matching).

**Additional file 5: Figure S3.** Cumulative incidence of heart failure hospitalization among patients without diabetes complication (before and after matching).

**Additional file 6: Table S3.** Heart failure hospitalization—sensitivity analysis by medication exposure and outcome follow up.

## Abbreviations

aHR: adjusted hazard ratio
CI: confidence interval
DPP4: dipeptidyl peptidase-4
EMPA-REG OUTCOME: empagliflozin removal of excess glucose outcome trial
EXAMINE: examination of cardiovascular outcomes with alogliptin versus standard of care
GLP-1: glucagon-like peptide-1
HR: hazard ratio
ICD-9, ICD-10: *International Classification of Diseases, Ninth Revision; International Classification of Diseases, Tenth Revision*
IPTW: inverse probability of treatment weighting
MSM: marginal structural modeling
OAD: oral antidiabetic drug
SAVOR-TIMI 53: saxagliptin assessment of vascular outcomes recorded in patients with diabetes mellitus-thrombolysis in myocardial infarction 53
SGLT2: sodium–glucose co-transporter 2
T2DM: type 2 diabetes mellitus
TECOS: trial evaluating cardiovascular outcomes with sitagliptin
