# Supplementary material for: Heart failure hospitalization risk associated with use of two classes of oral antidiabetic medications: an observational, real-world analysis
Source: Cardiovasc Diabetol. 2017 Jul 31;16:93. doi: 10.1186/s12933-017-0575-x (PMC5535291; doi:10.1186/s12933-017-0575-x)
Supplement: Supplementary file 1 — Additional file 1: Table S1. Breakdown of individual agents in each drug class. [file 12933_2017_575_MOESM1_ESM.docx]

**Additional Table S1: Breakdown of Individual Agents in Each Drug Class**

|  | **Overall Matched Cohort** | | **Matched Cohort** | |
| --- | --- | --- | --- | --- |
| **Agent** | **Total Patients** | **Patients With Heart Failure Hospitalization, n (%)** | **Total Patients** | **Patients With Heart Failure Hospitalization, n (%)** |
| DPP4 Inhibitors |  |  |  |  |
| Sitagliptin | 23,858 | 1,297 (5.4%) | 7,185 | 219 (3.0%) |
| Linagliptin | 3,831 | 193 (5.0%) | 1,272 | 48 (3.8%) |
| Saxagliptin | 3,811 | 233 (6.1%) | 1,154 | 38 (3.3%) |
| Alogliptin | 560 | 11 (2.0%) | 187 | 2 (1.1%) |
| SGLT2 Inhibitors |  |  |  |  |
| Canagliflozin | 3,894 | 89 (2.3%) | 3,490 | 79 (2.3%) |
| Dapagliflozin | 1,206 | 17 (1.4%) | 1,083 | 14 (1.3%) |
| Empagliflozin | 367 | 3 (0.8%) | 326 | 3 (0.9%) |
