# Supplementary figures and images for: Heart failure hospitalization risk associated with use of two classes of oral antidiabetic medications: an observational, real-world analysis
Source: Cardiovasc Diabetol. 2017 Jul 31;16:93. doi: 10.1186/s12933-017-0575-x (PMC5535291; doi:10.1186/s12933-017-0575-x)

**Additional Figure S1. Visual Assessment of Propensity Scores – Before and After Matching**

**
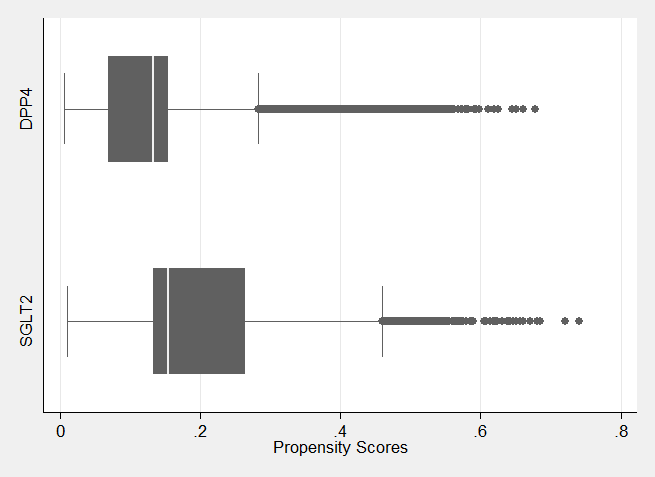
**

**
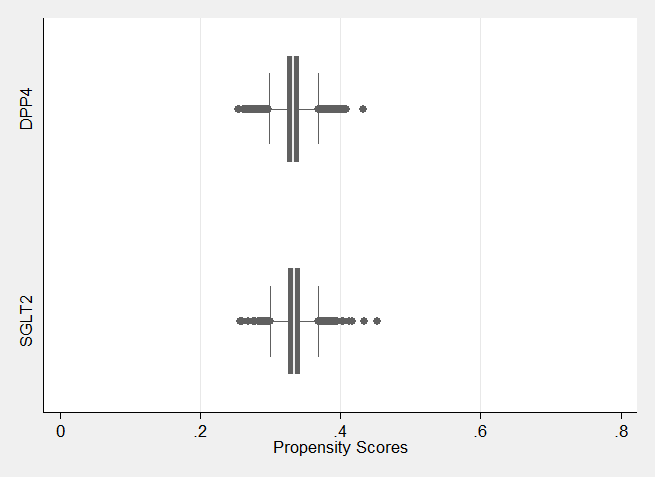
**

Supplement: Supplementary file 2 — Additional file 2: Figure S1. Visual assessment of propensity scores—before and after matching. [file 12933_2017_575_MOESM2_ESM.docx]
