# Supplementary material for: Heart failure hospitalization risk associated with use of two classes of oral antidiabetic medications: an observational, real-world analysis
Source: Cardiovasc Diabetol. 2017 Jul 31;16:93. doi: 10.1186/s12933-017-0575-x (PMC5535291; doi:10.1186/s12933-017-0575-x)
Supplement: Supplementary file 3 — Additional file 3: Table S2. Heart failure hospitalization—subgroup analysis by age and history of cardiovascular disease. [file 12933_2017_575_MOESM3_ESM.docx]

**Additional Table S2. Heart Failure Hospitalization – Subgroup Analysis by Age and History of Diabetes Complication**

|  | | | **Overall Unmatched Cohorts** | | **Matched Cohorts** | |
| --- | --- | --- | --- | --- | --- | --- |
|  | | | **DPP4** | **SGLT2** | **DPP4** | **SGLT2** |
| **By Age** | **Age ≥65** | Total patients | 10,050 | 722 | 1,444 | 722 |
|  |  | Patients with heart failure hospitalization, n (%) | 1,269 (12.6) | 34 (4.7) | 132 (9.1) | 34 (4.7) |
|  |  | HR (95% CI); Ref=DPP4 | 0.42 (0.30 – 0.59); p<.001 | | 0.60 (0.41 – 0.87); p=.008 | |
|  | **Age <65** | Total patients | 22,010 | 4,745 | 8,348 | 4,174 |
|  |  | Patients with heart failure hospitalization, n (%) | 465 (2.1) | 75 (1.6) | 162 (1.9) | 58 (1.4) |
|  |  | HR (95% CI); Ref=DPP4 | 0.80 (0.63 – 1.02); p=.07 | | 0.78 (0.57 – 1.05); p=.09 | |
| **Diabetes complication** | **Yes** | Total patients | 12,816 | 1,784 | 3,048 | 1,524 |
|  |  | Patients with heart failure hospitalization, n (%) | 1,448 (11.3) | 76 (4.3) | 210 (6.9) | 68 (4.5) |
|  |  | HR (95% CI); Ref=DPP4 | 0.39 (0.31 – 0.49) ; p<.001 | | 0.68 (0.52 – 0.90); p=.006 | |
|  | **No** | Total patients | 19,244 | 3,683 | 6,710 | 3,355 |
|  |  | Patients with heart failure hospitalization, n (%) | 286 (1.5) | 33 (0.9) | 81 (1.2) | 29 (0.9) |
|  |  | HR (95% CI), Ref=DPP4 | 0.68 (0.48 – 0.98); p=.04 | | 0.83 (0.54 – 1.27); p=.40 | |
| CI=confidence interval; DPP4= dipeptidyl peptidase-4; HR=hazard ratio; SGLT2=sodium-glucose co-transporter 2 | | | | | | |
