# Supplementary material for: Heart failure hospitalization risk associated with use of two classes of oral antidiabetic medications: an observational, real-world analysis
Source: Cardiovasc Diabetol. 2017 Jul 31;16:93. doi: 10.1186/s12933-017-0575-x (PMC5535291; doi:10.1186/s12933-017-0575-x)
Supplement: Supplementary file 4 — Additional file 4: Figure S2. Cumulative incidence of heart failure hospitalization among patients younger than 65 years (before and after matching). [file 12933_2017_575_MOESM4_ESM.docx]

**Additional Figure S2: Cumulative Incidence of Heart Failure Hospitalization Among Patients Younger Than 65 Years (Before and After Matching)**
