# Supplementary material for: Heart failure hospitalization risk associated with use of two classes of oral antidiabetic medications: an observational, real-world analysis
Source: Cardiovasc Diabetol. 2017 Jul 31;16:93. doi: 10.1186/s12933-017-0575-x (PMC5535291; doi:10.1186/s12933-017-0575-x)
Supplement: Supplementary file 6 — Additional file 6: Table S3. Heart failure hospitalization—sensitivity analysis by medication exposure and outcome follow up. [file 12933_2017_575_MOESM6_ESM.docx]

**Additional Table S3. Heart Failure Hospitalization – Sensitivity Analysis by Medication Exposure and Outcome Follow Up**

|  |  | **Overall Unmatched Cohorts** | | **Matched Cohorts** | |
| --- | --- | --- | --- | --- | --- |
|  |  | **DPP4** | **SGLT2** | **DPP4** | **SGLT2** |
| **≥ 90 Days Exposure** | Total patients | 24,337 | 4,219 | 7,308 | 3,654 |
|  | Patients with heart failure hospitalization, n (%) | 1,086 (4.5) | 73 (1.7) | 182 (2.5) | 61 (1.7) |
|  | HR (95% CI); Ref=DPP4 | 0.43 (0.34 – 0.54); p<.001 | | 0.74 (0.55 – 0.98); p=.038 | |
| **Post-Index Enrollment ≥24 Months** | Total patients | 16,603 | 2,432 | 4,382 | 2,191 |
|  | Patients with heart failure hospitalization, n (%) | 1,058 (6.4) | 68 (2.8) | 180 (4.1) | 60 (2.7) |
|  | HR (95% CI); Ref=DPP4 | 0.47 (0.36 – 0.59); p<.001 | | 0.72 (0.54 – 0.97); p=.030 | |
| CI=confidence interval; DPP4= dipeptidyl peptidase-4; HR=hazard ratio; SGLT2=sodium-glucose co-transporter 2 | | | | | |
